# Preliminary validity of the Draw a Shape Test for upper extremity assessment in multiple sclerosis

**DOI:** 10.1002/acn3.51705

**Published:** 2022-12-23

**Authors:** Jennifer S. Graves, Marco Ganzetti, Frank Dondelinger, Florian Lipsmeier, Shibeshih Belachew, Corrado Bernasconi, Xavier Montalban, Johan van Beek, Michael Baker, Christian Gossens, Michael Lindemann

**Affiliations:** ^1^ Department of Neurosciences University of California San Diego San Diego California USA; ^2^ F. Hoffmann‐La Roche Ltd Basel Switzerland; ^3^ Department of Neurology‐Neuroimmunology, Centre d'Esclerosi Múltiple de Catalunya (Cemcat) Hospital Universitari Vall d'Hebron Barcelona Spain

## Abstract

**Objective:**

To validate the smartphone sensor‐based Draw a Shape Test – a part of the Floodlight Proof‐of‐Concept app for remotely assessing multiple sclerosis‐related upper extremity impairment by tracing six different shapes.

**Methods:**

People with multiple sclerosis, classified functionally normal/abnormal via their Nine‐Hole Peg Test time, and healthy controls participated in a 24‐week, nonrandomized study. Spatial (trace accuracy), temporal (mean and variability in linear, angular, and radial drawing velocities, and dwell time ratio), and spatiotemporal features (trace celerity) were cross‐sectionally analyzed for correlation with standard clinical and brain magnetic resonance imaging (normalized brain volume and total lesion volume) disease burden measures, and for capacity to differentiate people with multiple sclerosis from healthy controls.

**Results:**

Data from 69 people with multiple sclerosis and 18 healthy controls were analyzed. Trace accuracy (all shapes), linear velocity variability (circle, figure‐of‐8, spiral shapes), and radial velocity variability (spiral shape) had a mostly fair/moderate‐to‐good correlation (|*r*| = 0.14–0.66) with all disease burden measures. Trace celerity also had mostly fair/moderate‐to‐good correlation (|*r*| = 0.18–0.41) with Nine‐Hole Peg Test performance, cerebellar functional system score, and brain magnetic resonance imaging. Furthermore, partial correlation analysis related these results to motor impairment. People with multiple sclerosis showed greater drawing velocity variability, though slower mean velocity, than healthy controls. Linear velocity (spiral shape) and angular velocity (circle shape) potentially differentiate functionally normal people with multiple sclerosis from healthy controls.

**Interpretation:**

The Draw a Shape Test objectively assesses upper extremity impairment and correlates with all disease burden measures, thus aiding multiple sclerosis‐related upper extremity impairment characterization.

## Introduction

Multiple sclerosis (MS) is a chronic inflammatory, autoimmune, demyelinating disease of the central nervous system, which can cause impairment to several functional domains.^1^ One commonly affected domain is upper extremity function, in which impairment arises from either ataxia, motor dysfunction, or sensory dysfunction.^2^, ^3^, ^4^ Reports have suggested that 60–76% of people with MS (PwMS) experience or show signs of impaired upper extremity function.^5^, ^6^, ^7^

Upper extremity dysfunction can impact quality of life and daily activities including, but not limited to, grasping and manipulating objects, typing, and handwriting.^4^, ^8^, ^9^, ^10^ Notwithstanding, functional ability, including upper extremity function, is not routinely assessed in clinical practice with disability scales.^11^ Currently, despite the availability of other tools, the Nine‐Hole Peg Test (9HPT) is the only well‐accepted standardized test assessing upper extremity function.^12^ The relationships between 9HPT and MRI have been previously explored in PwMS, yielding substantial trends and correlations.^13^ A 20% increase in the 9HPT time is commonly used to define a clinically meaningful worsening.^12^ However, the 9HPT has several key limitations, such as the need for patient supervision and pegboard, in addition to the low granularity and functionality of the summary score of overall performance, limited to reporting the time per hand. Developing accessible, objective measures that capture upper extremity dysfunction and progression in MS is an unmet need. Early evidence has shown the potential of novel, smartphone sensor‐based, visually guided tasks to objectively assess upper extremity function with high granularity in MS,^3^, ^14^, ^15^, ^16^, ^17^ Parkinson's disease,^18^, ^19^ Huntington's disease,^20^ and essential tremor.^21^

The study “Monitoring of Multiple Sclerosis Participants With the Use of Digital Technology (Smartphones and Smartwatches) – A Feasibility Study” was the first clinical trial to implement the Draw a Shape (DaS) Test from the Floodlight Proof‐of‐Concept (PoC) app.^22^ The DaS Test has also been used as a remote, smartphone sensor‐based assessment of upper extremity function in Huntington's disease^20^ and Parkinson's disease.^18^ We previously showed that two test features, overall mean trace accuracy and overall mean trace celerity, had good test–retest reliability in PwMS as measured by intraclass coefficient correlation (second model, first class)^23^ (ICC[2,1]: 0.85 [0.79–0.90] and 0.81 [0.73–0.87], respectively) and showed fair correlations with 9HPT (*r* = −0.48 and *r* = −0.40; *p* < 0.001) and T2‐weighted fluid‐attenuated inversion recovery (T2‐FLAIR) lesion volume (both *r* = −0.26; *p* <0.05).^16^ Notably, the DaS Test offers a multidimensional feature space (i.e., a set of measures derived from the DaS Test that capture distinctly different aspects of upper extremity function, e.g., spatial, temporal, and spatiotemporal aspects [mathematical features of the raw sensor data waveform]), which may address one of 9HPT's key limitations by providing a more granular assessment of upper extremity function. Additionally, recent qualitative data suggest face and ecological validity (i.e., the degree to which the test achieves its intended aims and reflects the level of performance of daily activities), with PwMS who performed the DaS Test identifying dexterity, hand steadiness, and focus as key skills required for test performance and daily activities (e.g., writing, coloring, drawing) that are affected by MS.^24^, ^25^

Here we present our secondary, exploratory analyses that expand on prior work by increasing the feature space to include spatial, spatiotemporal, and temporal features, including shape‐specific features, and investigate the use of the DaS Test as a convenient and objective assessment of upper extremity function. Using this expanded feature set, we assess the DaS Test's agreement with the clinical and brain MRI measures of MS disease state, and its ability to differentiate PwMS, including those with normal 9HPT times, from healthy controls (HC). We also analyze all features applicable to each shape with detailed comparisons.

## Methods

### Study design and participants

This 24‐week, nonrandomized study assessed the feasibility of remotely monitoring PwMS with the Floodlight PoC app on a provisioned smartphone. The full study design, and inclusion and exclusion criteria have been previously reported.^22^ Seventy‐six PwMS and 25 HC aged 18–55 years were enrolled. The HC were often the partners of PwMS, and while not matched, there were only small differences in age. Seventy PwMS were considered adequate to detect a linear correlation coefficient of 0.33 with >80% power. PwMS were diagnosed using the 2010 revised McDonald criteria^26^ and had a baseline Expanded Disability Status Scale (EDSS) score^27^ of 0.0–5.5. Within the PwMS group, participants were further subclassified as PwMS with normal (PwMS‐Normal) and abnormal 9HPT times at baseline (PwMS‐Abnormal); an abnormal 9HPT time was greater than the mean plus two standard deviations of the normative data of HC derived from Erasmus et al.^3^ Thresholds were 22.58 sec for both hands combined (mean of both hands), 22.15 sec for the dominant hand, and 23.01 sec for the nondominant hand.

**Figure 1:**
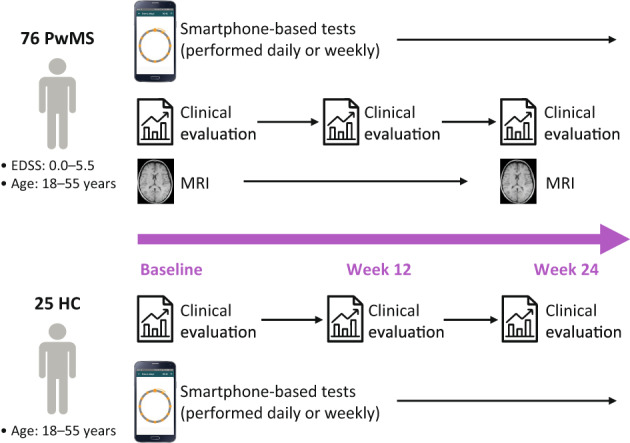
Study design. The Floodlight PoC app prompted both PwMS and HC to perform daily and weekly smartphone‐based tests on a provisioned Samsung Galaxy S7 smartphone. This included the daily Draw a Shape Test. Additionally, PwMS and HC were clinically assessed at the three clinic visits at baseline, week 12, and week 24 (end of study). These assessments included, among others, the 9HPT, oral SDMT, MSIS‐29 (administered in PwMS only), and EDSS (administered in PwMS only). Brain MRI was collected from PwMS at baseline and week 24. 9HPT, Nine‐Hole Peg Test; EDSS, Expanded Disability Status Scale; HC, healthy controls; MSIS‐29, 29‐item Multiple Sclerosis Impact Scale; PoC, Proof‐of‐Concept; PwMS, people with multiple sclerosis; SDMT, Symbol Digit Modalities Test.

At every scheduled clinical visit (baseline, week 12, and week 24), both PwMS and HC were clinically evaluated with the 9HPT and the oral Symbol Digit Modalities Test (SDMT; Fig. 1). Additionally, PwMS were assessed by the 29‐item Multiple Sclerosis Impact Scale (MSIS‐29)^28^ and EDSS at all three clinical visits, and by MRI at baseline and week 24. All Floodlight tests (except the electronic SDMT [e‐SDMT]) were performed daily^22^ to improve data granularity. The e‐SDMT was administered weekly to reduce the impact of practice effects commonly observed on the SDMT.^29^, ^30^ At the baseline visit, all participants received a Samsung Galaxy S7 smartphone with the Floodlight PoC app pre‐installed. The app instructed the participants to perform daily and weekly smartphone sensor‐based active tests to assess their upper extremity function, cognition, gait, and balance.

### Standard protocol approvals, registrations, and patient consents

All participants provided informed consent, and ethical approval was obtained from the ethics committee of the Hospital Universitari Vall d'Hebron, Barcelona, Spain (study number: PR(AG)300/2016) and the Institutional Review Board of the University of California San Francisco, San Francisco, CA, USA (reference number: 175728) prior to study initiation. The study was registered on clinicaltrials.gov (NCT02952911).

### The Draw a Shape Test

**Figure 2:**
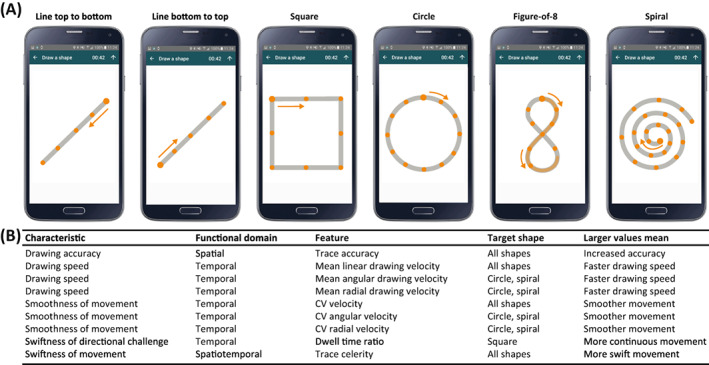
The Draw a Shape Test. (A) Participants were instructed to draw six different shapes of increasing complexity by tracing the reference shape with their finger on the smartphone display as fast and as accurately as possible. (B) Spatial, temporal, and spatiotemporal features were computed for each of the six shapes. In addition, shape‐specific features were derived from the circle and spiral (mean and CV angular velocity; mean and CV radial velocity) as well as the square (dwell time ratio). CV, coefficient of variation.

The DaS Test is a remotely performed, smartphone sensor‐based, active test of upper extremity function, which is included in the Floodlight PoC app and is performed daily.^14^ To perform the DaS Test, all study participants held their smartphones in the untested hand and traced six increasingly complex shapes on the touchscreen (two diagonal lines [one from the top right corner to the bottom left corner and one from the bottom left corner to the top right corner], a square, a circle, a figure‐of‐8, and a spiral, all drawn clockwise) with the index finger of the tested hand “as fast and as accurately as possible” (Fig. 2). To draw a shape successfully, the participants had to continuously slide their finger on the touchscreen and connect indicated start and endpoints while passing through all indicated checkpoints within 30 sec. The dominant and nondominant hands were tested on alternate test runs. Each participant had two attempts per shape for successful completion; the second attempt was displayed only if the first attempt to draw the shape was not successful.

### Feature extraction

Spatial, temporal, and spatiotemporal waveform features were extracted for each shape (Fig. 2, Fig. S1A).^14^, ^16^ Spatial features included trace accuracy, defined as the proportion of sample points that overlapped with the reference shape, which was computed for all six shapes. Temporal performance was assessed by features capturing drawing velocity and swiftness in directional challenge. Velocity‐based features included the mean and coefficient of variation (CV) of linear drawing velocity (Fig. S1B). These features were computed for all shapes. Additionally, shape‐specific features were computed for both the circle and spiral (mean and CV angular velocity [i.e., how fast the fingertip rotates with respect to the center of the shape]; mean and CV radial velocity [i.e., the rate of change at which the fingertip moves directly away from the center of the shape]; Fig. S1C) as well as the square (dwell time ratio; Fig. S1D). The dwell time ratio describes the time spent at the three corners when drawing the square relative to the total drawing duration. Finally, spatiotemporal features included trace celerity, which was computed for all shapes by dividing trace accuracy by the total drawing duration.

### Data processing

As the DaS Test was performed unsupervised, data quality measures were implemented to ensure test performance was representative of upper extremity function. Invalid test runs were identified by quality control flags, which detected test runs that were executed without any screen interactions or test runs characterized by “play‐to‐quit” attempts (defined as test runs having both short drawing duration [<0.5 sec] and low trace accuracy [<10% overlap with the reference shape]) and were subsequently disregarded from the analyses. All remaining test runs, that is all valid test runs, contributed by a participant were aggregated by taking the median across the entire study duration as in Montalban et al.^16^ Additionally, the mean of the three in‐clinic assessments (or mean of two assessments for MRI) was computed for the correlation analysis.

### Statistical analysis

All participants with at least 14 valid test runs were included in the analyses. Based on a characteristics comparison between the analyzed population (see Table 1) versus those of the total population reported by Midaglia et al.,^22^ 14 valid test runs were calculated as optimal for a stable cross‐sectional analysis. This is because including <14 valid tests may not be indicative of the true functional ability of PwMS, whereas including >14 valid tests may be biased towards a less impaired sample. For the correlation analysis, the DaS Test features were correlated against the 9HPT, oral SDMT, MSIS‐29 (hand items 2, 3, 8–10, 15), EDSS (including cerebellar functional system and pyramidal functional system), and MRI measures (normalized brain volume and T2‐FLAIR lesion volume). The strength of the correlation was considered not correlated (|*r*| < 0.25), fair (|*r*| = 0.25–0.49), moderate‐to‐good (|*r*| = 0.50–0.75), or good‐to‐excellent (|*r*| > 0.75).^31^ Additionally, a partial correlation analysis was conducted by correlating each DaS Test feature against 9HPT while adjusting for oral SDMT and against oral SDMT while adjusting for 9HPT. A separate analysis examined the DaS Test's ability to differentiate among PwMS, PwMS‐Normal, PwMS‐Abnormal, and HC. Differences between the groups were assessed for statistical significance by Mann–Whitney *U* test; statistical significance was set at *p* < 0.05 without correction for multiple comparisons, as the analyses were exploratory rather than confirmatory. Data from the first attempt were used to compare like with like in all analyses. Furthermore, all analyses were adjusted for age and sex with a robust linear model,^32^ and were conducted for both hands combined (mean of both hands) and for the individual hands.

**Table 1:** Baseline demographics and disease characteristics.

Variable	Assessed study population (*n* = 87)^1^	
	HC (*n* = 18)	PwMS (*n* = 69)
Female, *n* (%)	6 (33)	47 (68)
Age, years, mean ± SD	35.0 ± 8.9	39.4 ± 7.8
Height, cm, mean ± SD	174.0 ± 7.7	169.1 ± 9.0
Diagnosis, *n* (%)		
Relapsing–remitting multiple sclerosis	–	62 (90)
Primary progressive multiple sclerosis	–	3 (4)
Secondary progressive multiple sclerosis	–	4 (6)
Disease onset from baseline, years, mean ± SD	–	9.1 ± 6.5
Oral SDMT, mean ± SD (range)	64.6 ± 8.4 (52.0–77.0)	53.7 ± 12.0 (26.0–77.0)
Expanded Disability Status Scale, mean ± SD (range)		
Score	–	2.43 ± 1.36 (0.0–5.5)
Cerebellar Functional System	–	0.78 ± 0.92 (0.0–3.0)
Pyramidal Functional System	–	1.36 ± 1.06 (0.0–3.0)
Nine‐Hole Peg Test time, sec, mean ± SD		
Both hands combined	18.83 ± 1.72	22.29 ± 4.15
Dominant hand	18.66 ± 1.95	22.13 ± 4.70
Nondominant hand	19.01 ± 1.79	22.46 ± 4.38
29‐item Multiple Sclerosis Impact Scale hand items, mean ± SD^2^	–	24.56 ± 24.58
Total brain volume, mL, mean ± SD	–	1,470.9 ± 76.2
T2‐FLAIR lesion volume, mL, mean ± SD	–	6.3 ± 7.6

FLAIR, fluid‐attenuated inversion recovery; HC, healthy control; PwMS, people with multiple sclerosis; SDMT, Symbol Digit Modalities Test; T2, T2‐weighted.

^1^ All study participants who fulfilled the quality control criteria were included in the assessed study population.

^2^ The hand‐related components include items 2, 3, 8, 9, 10, and 15.

## Results

In total, 76 PwMS and 25 HC were enrolled between November 28, 2016 and May 4, 2018 (end of study). Following the quality control, valid test runs were available for 69 (91%) PwMS and 18 (72%) HC. Baseline demographics and disease characteristics of the assessed study population (Table 1) were similar to those previously reported by Midaglia et al.^22^ for the entire study population. PwMS included in the analyses mostly presented mild disease characteristics with limited upper extremity dysfunction, having a mean EDSS score of 2.43 (range: 0.0–5.5), and a mean 9HPT time of 22.29 sec (SD: 4.15). Furthermore, changes observed for the 9HPT, EDSS, and MRI (T2‐FLAIR lesion volume) measures between the baseline and the end of study (week 24) clinical visit were mostly within one standard deviation of the baseline assessments with no change over time observed (Fig. S2).

### Correlation with standard clinical scales and brain MRI measures of MS disease state

**Figure 3:**
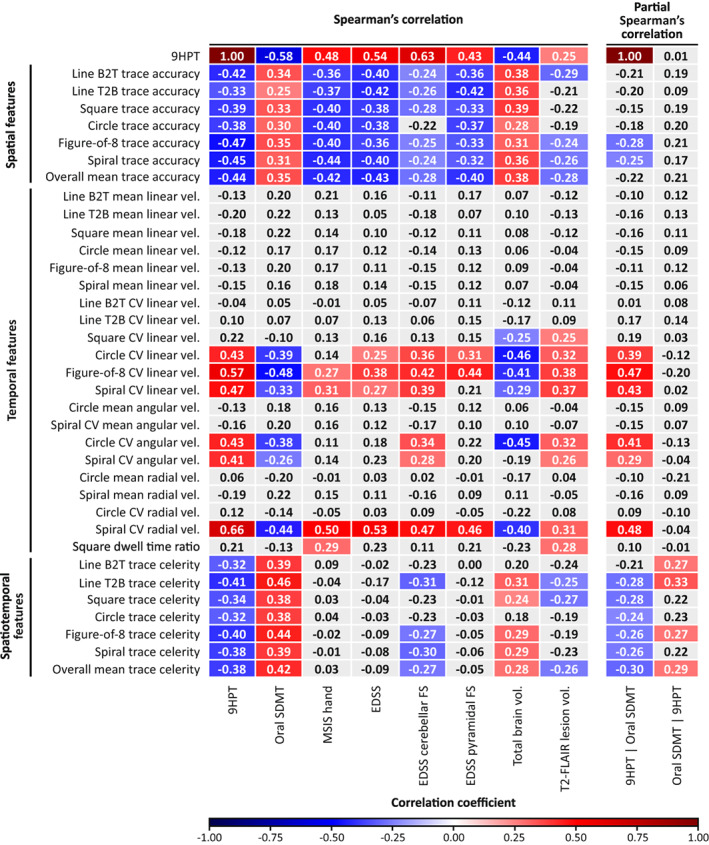
Spearman's rank correlations and partial Spearman's correlations of the DaS Test (for both hands combined) with standard clinical and MRI measures in PwMS. Correlation coefficients are shown. Statistically significant positive correlations are highlighted in red, and statistically significant negative correlations are in blue. Partial correlations included correlations between the DaS Test and 9HPT after adjusting for oral SDMT (9HPT | oral SDMT) and between the DaS Test and oral SDMT after adjusting for 9HPT (Oral SDMT | 9HPT). The strengths of the correlations were either fair (|*r*| = 0.25–0.49) or moderate‐to‐good (|*r*| = 0.50–0.75). Higher values indicated better performance on features assessing trace accuracy, mean velocity, and trace celerity. Lower values indicated better performance on features assessing CV velocity and dwell time ratio. 9HPT, Nine‐Hole Peg Test; B2T, bottom to top; CV, coefficient of variation; DaS, Draw a Shape; EDSS, Expanded Disability Status Scale; FLAIR, fluid‐attenuated inversion recovery; FS, functional system; MSIS, Multiple Sclerosis Impact Scale; PwMS, people with multiple sclerosis; SDMT, Symbol Digit Modalities Test; T2, T2‐weighted; T2B, top to bottom; vel., drawing velocity.

The correlations between digital features obtained for both hands combined and standard clinical scales as well as brain MRI measures of MS disease state (9HPT: |*r*| = 0.32–0.66, oral SDMT: |*r*| = 0.25–0.48, MSIS‐29 hand items: |*r*| = 0.27–0.50, EDSS: |*r*| = 0.25–0.53, cerebellar functional system: |*r*| = 0.24–0.47, pyramidal functional system: |*r*| = 0.31–0.46, total brain volume: |*r*| = 0.24–0.46, T2 lesion volume: |*r*| = 0.24–0.38, for *p* < 0.05) are summarized in Figure 3. Worse performance captured by spatial features was associated with worse outcomes on standard clinical scales and MRI outcomes, showing fair correlations with 9HPT (*r* = −0.33 to −0.47), oral SDMT (*r* = 0.25–0.35), MSIS‐29 hand items (*r* = −0.36 to −0.44), EDSS (*r* = −0.36 to −0.43), pyramidal functional system (*r* = −0.32 to −0.42), and total brain volume (*r* = 0.28–0.39). For most shapes, spatial features also showed mostly fair correlations with cerebellar functional system score (*r* = −0.24 to −0.28) and T2 lesion volume (*r* = −0.24 to −0.29), for *p* < 0.05. In the temporal domain, greater variability (CV) in drawing velocity, but not slower mean drawing velocity, was generally related to worse outcomes on all standard clinical scales and MRI measures when considering the complex, round shapes. For most of these features, correlations with 9HPT (*r* = 0.41–0.66), MSIS‐29 hand items (*r* = 0.27–0.50), EDSS (*r* = 0.25–0.53) were fair or moderate‐to‐good in strength, and correlations with oral SDMT (*r* = −0.26 to −0.48), cerebellar function system (*r* = 0.28–0.47), pyramidal functional system (*r* = 0.31–0.46), total brain volume (*r* = −0.25 to −0.46), and T2 lesion volume (*r* = 0.25–0.38) reached fair strength, for *p* < 0.05. Worse spatiotemporal performance was associated with worse outcomes on the 9HPT (*r* = −0.32 to –0.41) and oral SDMT (*r* = 0.38–0.46) and, on most shapes, with cerebellar functional system score (*r* = −0.27 to −0.31), total brain volume (*r* = 0.24–0.31), and T2 lesion volume (*r* = −0.25 to −0.27), for *p* < 0.05. These correlations were all mostly fair in strength.

Few differences between shape‐specific features were noted. While CV angular velocity on both the circle and spiral correlated with 9HPT (*r* = 0.43 and *r* = 0.41, respectively), cerebellar functional system score (*r* = 0.34 and *r* = 0.28, respectively), and T2 lesion volume (*r* = 0.32 and *r* = 0.26, respectively), CV radial velocity on the spiral, but not on the circle, showed moderate‐to‐good correlations with the 9HPT (*r* = 0.66), MSIS‐29 hand items (*r* = 0.50), and EDSS (*r* = 0.53), and fair correlations with all other clinical scales and MRI outcome (oral SDMT: *r* = −0.44, cerebellar functional system score: *r* = 0.47, pyramidal functional system score: *r* = 0.46, total brain volume: *r* = −0.40, T2 lesion volume: *r* = 0.31). Furthermore, a higher square dwell time ratio was associated with worse outcomes on the MSIS‐29 hand items (*r* = 0.29; fair strength) and with larger T2 lesion volume (*r* = 0.28; fair strength).

The partial correlation analysis revealed that correlations with 9HPT remained statistically significant after adjusting for oral SDMT for CV linear velocity on the round shapes (*r* = 0.39–0.47), CV angular velocity on the circle (*r* = 0.41), and CV radial velocity on the spiral (*r* = 0.48). However, the correlations between these features and oral SDMT were no longer statistically significant after adjusting for 9HPT (all *p* > 0.05).

In an analysis of individual hand performance (Table S1), results were predominantly comparable for spatial and temporal features. However, for more complex spatiotemporal features, correlations were associated with hand dominance. Correlations between these features with cerebellar functional system score, brain volume, or lesion volume were stronger on the dominant hand. By comparison, correlations between these spatiotemporal features and 9HPT were generally stronger on the nondominant hand.

### Ability to differentiate between HC and PwMS

The comparison between PwMS, PwMS‐Abnormal, and PwMS‐Normal subgroups, and HC for both hands combined are summarized in Table 2. Compared with HC, PwMS drew the complex, round shapes with slower linear velocity and increased variability in linear velocity (all *p* < 0.05 except for mean linear velocity on the circle). PwMS also performed worse than HC on spatiotemporal features on both simple, linear shapes, and complex, round shapes (all *p* < 0.05 except line top to bottom). Additionally, statistically significant differences were observed between PwMS‐Abnormal and either HC or PwMS‐Normal on features of all three categories (spatial, spatiotemporal, and temporal).

**Table 2:** Descriptive statistics by subject group (both hands combined).

Feature	Mean ± SD				*p* ^1^			
	HC (*n* = 18)	PwMS (*n* = 69)	PwMS‐Normal (*n* = 51)	PwMS‐Abnormal (*n* = 18)	HC vs. PwMS	HC vs. PwMS‐Normal	HC vs. PwMS‐Abnormal	PwMS‐Normal vs. PwMS‐Abnormal
Spatial features								
*Trace accuracy*								
Line bottom to top	0.93 ± 0.08	0.90 ± 0.10	0.92 ± 0.09	0.85 ± 0.10	0.457	0.924	**0.014**	**0.007**
Line top to bottom	0.88 ± 0.05	0.85 ± 0.10	0.87 ± 0.09	0.79 ± 0.10	0.258	0.848	**0.003**	**0.006**
Square	0.92 ± 0.05	0.89 ± 0.09	0.91 ± 0.09	0.86 ± 0.08	0.457	0.924	**0.043**	**0.028**
Circle	0.93 ± 0.06	0.90 ± 0.10	0.91 ± 0.09	0.86 ± 0.10	0.396	0.870	**0.029**	**0.022**
Figure‐of‐8	0.93 ± 0.07	0.91 ± 0.10	0.93 ± 0.09	0.85 ± 0.10	0.463	0.891	**0.011**	**0.005**
Spiral	0.89 ± 0.06	0.86 ± 0.09	0.88 ± 0.08	0.81 ± 0.10	0.340	0.924	**0.008**	**0.008**
Mean overall trace accuracy	0.89 ± 0.06	0.86 ± 0.09	0.88 ± 0.09	0.81 ± 0.09	0.285	0.827	**0.007**	**0.007**
Temporal features								
*Mean linear velocity, mm/sec*								
Line bottom to top	49.5 ± 16.4	44.0 ± 32.3	47.9 ± 34.8	33.0 ± 20.9	0.088	0.280	**0.008**	**0.016**
Line top to bottom	48.9 ± 14.6	45.3 ± 29.2	49.1 ± 30.1	34.3 ± 24.0	0.180	0.652	**0.003**	**0.007**
Square	50.4 ± 7.3	44.0 ± 19.1	47.3 ± 19.4	34.7 ± 15.3	**0.031**	0.163	**0.001**	**0.005**
Circle	42.0 ± 8.5	37.9 ± 24.2	41.1 ± 25.7	28.7 ± 16.7	0.059	0.274	**0.002**	**0.011**
Figure‐of‐8	34.4 ± 7.1	29.5 ± 20.7	32.7 ± 22.1	20.3 ± 12.3	**0.015**	0.140	**<0.001**	**0.003**
Spiral	40.1 ± 7.5	36.1 ± 22.2	39.4 ± 23.4	26.7 ± 15.1	**0.032**	0.234	**<0.001**	**0.003**
*CV linear velocity*								
Line bottom to top	0.46 ± 0.06	0.49 ± 0.08	0.49 ± 0.08	0.48 ± 0.08	0.330	0.318	0.527	0.816
Line top to bottom	0.49 ± 0.07	0.51 ± 0.09	0.51 ± 0.09	0.53 ± 0.08	0.489	0.623	0.343	0.682
Square	0.53 ± 0.05	0.55 ± 0.09	0.54 ± 0.08	0.60 ± 0.11	0.438	0.827	0.066	**0.048**
Circle	0.33 ± 0.02	0.36 ± 0.04	0.34 ± 0.03	0.40 ± 0.05	**0.004**	0.065	**<0.001**	**<0.001**
Figure‐of‐8	0.35 ± 0.02	0.37 ± 0.04	0.36 ± 0.03	0.42 ± 0.05	**0.027**	0.374	**<0.001**	**<0.001**
Spiral	0.35 ± 0.03	0.39 ± 0.05	0.38 ± 0.03	0.44 ± 0.07	**<0.001**	**0.009**	**<0.001**	**<0.001**
*Mean angular velocity, rad/sec*								
Circle	1.55 ± 0.33	1.40 ± 0.92	1.53 ± 0.98	1.05 ± 0.62	0.058	0.299	**<0.001**	**0.007**
Spiral	2.44 ± 0.45	2.20 ± 1.34	2.41 ± 1.42	1.60 ± 0.88	**0.024**	0.204	**<0.001**	**0.002**
*CV angular velocity*								
Circle	0.32 ± 0.02	0.36 ± 0.05	0.34 ± 0.03	0.40 ± 0.06	**0.002**	**0.043**	**<0.001**	**<0.001**
Spiral	0.55 ± 0.04	0.54 ± 0.06	0.53 ± 0.05	0.57 ± 0.07	0.593	0.190	0.155	**0.010**
*Mean radial velocity, mm/sec*								
Circle	0.10 ± 0.25	0.14 ± 0.30	0.14 ± 0.33	0.14 ± 0.16	0.645	0.604	0.849	0.692
Spiral	3.53 ± 0.59	3.15 ± 1.78	3.46 ± 1.87	2.28 ± 1.13	**0.024**	0.229	**<0.001**	**<0.001**
*CV radial velocity*								
Circle	9.48 ± 8.32	8.62 ± 9.36	8.06 ± 9.58	10.20 ± 8.78	0.867	0.733	0.776	0.512
Spiral	1.96 ± 0.19	2.13 ± 0.45	1.97 ± 0.29	2.56 ± 0.55	0.300	0.672	**<0.001**	**<0.001**
*Dwell time ratio*								
Square	0.38 ± 0.04	0.38 ± 0.06	0.37 ± 0.05	0.40 ± 0.07	0.652	0.382	0.506	0.204
Spatiotemporal features								
*Trace celerity, 1/sec*								
Line bottom to top	0.66 ± 0.17	0.56 ± 0.30	0.62 ± 0.31	0.38 ± 0.18	**0.014**	0.129	**<0.001**	**<0.001**
Line top to bottom	0.57 ± 0.13	0.51 ± 0.27	0.58 ± 0.27	0.31 ± 0.16	0.078	0.642	**<0.001**	**<0.001**
Square	0.22 ± 0.03	0.18 ± 0.05	0.20 ± 0.05	0.14 ± 0.04	**0.004**	0.058	**<0.001**	**<0.001**
Circle	0.22 ± 0.03	0.19 ± 0.06	0.21 ± 0.06	0.14 ± 0.05	**0.007**	0.095	**<0.001**	**<0.001**
Figure‐of‐8	0.23 ± 0.03	0.19 ± 0.07	0.22 ± 0.07	0.13 ± 0.05	**0.003**	0.061	**<0.001**	**<0.001**
Spiral	0.13 ± 0.02	0.11 ± 0.04	0.13 ± 0.04	0.08 ± 0.03	**0.007**	0.123	**<0.001**	**<0.001**
Mean overall trace celerity	0.36 ± 0.06	0.31 ± 0.12	0.34 ± 0.12	0.22 ± 0.08	**0.013**	0.155	**<0.001**	**<0.001**

Higher values indicated better performance on features assessing trace accuracy, mean velocity, and trace celerity. Lower values indicated better performance on features assessing CV velocity and dwell time ratio. Statistically significant *p* values (<0.05) are in bold.

CV, coefficient of variation; HC, healthy control; PwMS, people with multiple sclerosis.

^1^ Mann–Whitney *U* test.

The analysis of shape‐specific features revealed that PwMS drew the spiral with slower angular and radial velocity than HC (both *p* < 0.05). Drawings of the spiral by PwMS‐Abnormal were also characterized by increased variability in angular velocity (compared with PwMS‐Normal; *p* < 0.05) and by increased variability in radial velocity (compared with either HC or PwMS‐Normal; both *p* < 0.001). Similarly on the circle, mean angular velocity differentiated between PwMS‐Abnormal and either HC (*p* < 0.001) or PwMS‐Normal (*p* < 0.01), while variability in angular velocity differentiated between PwMS and HC (*p* < 0.01). Dwell time ratio did not differentiate between groups (all *p* > 0.05).

**Figure 4:**
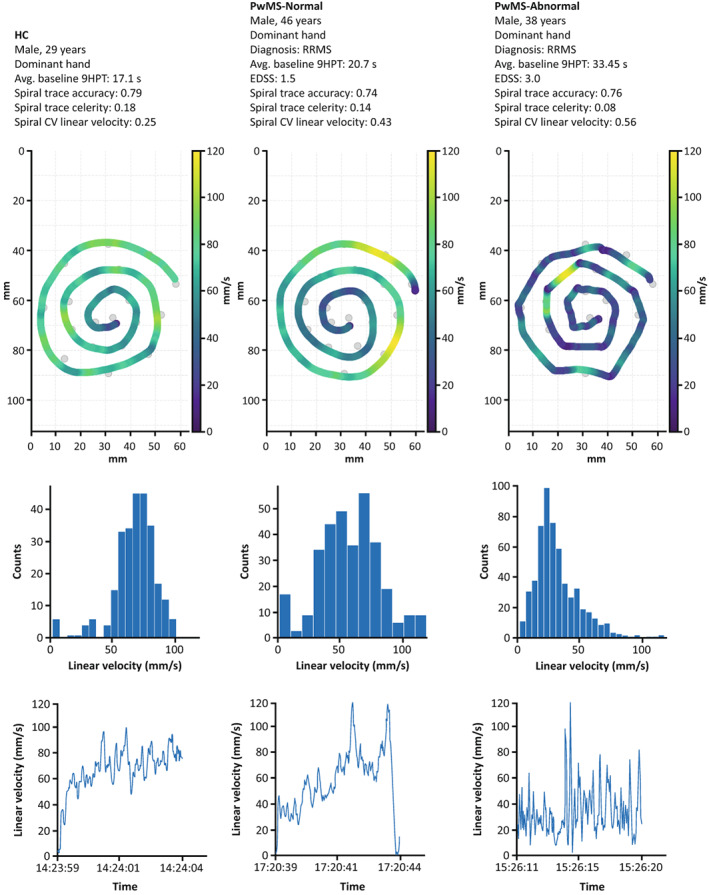
Increased variability in spiral linear velocity with higher levels of MS‐related impairment. The top row shows the drawn traces of the spiral shape for an HC (left), a PwMS‐Normal (middle), and a PwMS‐Abnormal (right), with the color bar indicating the linear velocity. The corresponding histograms and time series of the spiral linear velocity are shown in the middle and bottom rows. The *x*‐axis in the bottom row displays the time of day (h:min:sec). 9HPT, Nine‐Hole Peg Test; avg., average; CV, coefficient of variation; EDSS, Expanded Disability Status Scale; HC, healthy control; MS, multiple sclerosis; PwMS, people with multiple sclerosis; RRMS, relapsing–remitting multiple sclerosis; s, seconds.

**Figure 5:**
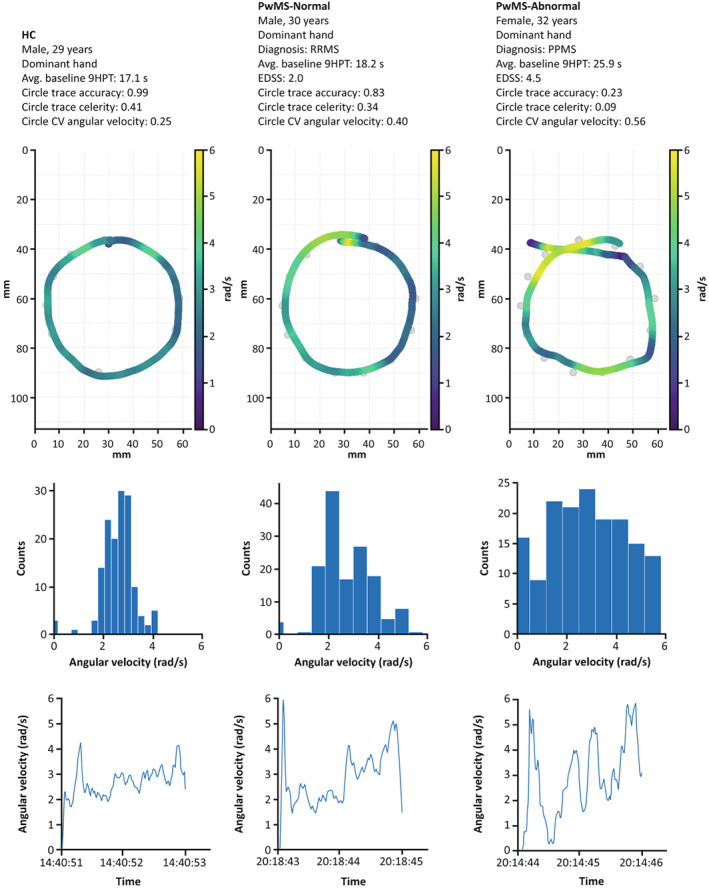
Increased variability in circle angular velocity with higher levels of MS‐related impairment. The top row shows the drawn traces of the circle shape for a HC (left), a PwMS‐Normal (middle), and a PwMS‐Abnormal (right), with the color bar indicating the angular velocity. The corresponding histograms and time series of the circle angular velocity are shown in the middle and bottom rows. The *x*‐axis in the bottom row displays the time of day (h:min:sec). 9HPT, Nine‐Hole Peg Test; avg., average; CV, coefficient of variation; EDSS, Expanded Disability Status Scale; HC, healthy control; MS, multiple sclerosis; PPMS, primary progressive multiple sclerosis; PwMS, people with multiple sclerosis; RRMS, relapsing–remitting multiple sclerosis; s, seconds.

Of note, CV linear velocity on the spiral (*p* < 0.01) and CV angular velocity on the circle (*p* < 0.05) were both increased in PwMS‐Normal versus HC despite PwMS‐Normal having no apparent upper extremity function impairment as measured by the 9HPT. Having observed the good performance of these variability‐based velocity features, we further visualized them by comparing the representative tests from people with increasing levels of impairment. These visualizations depict that increasing levels of impairment were associated with increasing variability in linear velocity on the spiral (Fig. 4), angular velocity on the circle (Fig. 5), and radial velocity on the spiral (Fig. S3). Furthermore, the circle angular velocity was characterized by both high‐speed and low‐speed drawing sections. While spiral radial velocity showed a similar pattern, the interpretation of these features differs as described in the Discussion.

The ability of the temporal features to differentiate PwMS from HC was similar for the individual hands and both hands combined. However, the ability of spatial features to differentiate between PwMS‐Abnormal and either HC or PwMS‐Normal tended to be greater on the nondominant hand than on the dominant hand, particularly on the simpler, linear shapes (Table S2).

## Discussion

Our results suggest that the remotely performed, smartphone sensor‐based DaS Test objectively assesses upper extremity function and may help accurately detect MS‐related functional impairment and reduce patient burden. The strengths of the DaS Test include its multidimensional feature characteristics, capturing spatial, temporal, and spatiotemporal information across six increasingly complex shapes. We previously showed that such multidimensionality is necessary to predict 9HPT time with a mean absolute error comparable to the variability of the actual 9HPT times.^14^ Here, we identified features that showed fair, reaching moderate‐to‐good, correlations with standard clinical scales and brain MRI measures (normalized brain volume and total lesion volume).

Overall mean trace celerity was designed to measure how accurately and quickly PwMS can draw the six shapes. This feature showed fair correlations with the 9HPT and differentiated both between HC and PwMS, and between PwMS‐Normal and PwMS‐Abnormal. Similar results were obtained when assessing trace celerity on the individual shapes, thereby corroborating these findings. This suggests that overall mean trace celerity fulfills two important criteria of an ideal DaS feature: (1) at least a fair agreement with standard clinical scales of upper extremity function and (2) the ability to differentiate between groups with different levels of upper extremity impairment. Additionally, we previously reported good test–retest reliability for this feature.^16^

The strongest associations with MS disease burden as measured by brain MRI measures and standard clinical scales of upper extremity function, cognitive information processing speed, and overall disease severity were observed for the spatial test features for all shapes and the variability‐based temporal features for the most complex shapes (circle, figure‐of‐8, and spiral). Notably, the pyramidal function system was associated with spatial test features across all shapes, including simple, linear shapes. Contrastingly, cerebellar ataxia, measured by the cerebellar functional system score of the EDSS, was generally associated with spatial, temporal, and spatiotemporal features. Previous studies have linked cerebellar ataxia to impaired motor coordination and increased variability in motor performance.^33^, ^34^

Correlations with standard clinical scales and brain MRI measures tended to be stronger for variability‐based drawing velocity features than for mean drawing velocity. In PwMS, keeping a low variability in drawing velocity features could rely more strongly on sensory feedback compared with the actual drawing velocity, which could potentially explicate the stronger association of the variability with impairment.^35^

Moreover, drawing variability features have been proposed as a measure of fine motor control.^35^ For a perfect spiral, the expected radial drawing velocity would be a positive value, as radial velocity measures the movement outward from the center. Hence, negative values indicate an acceleration in the wrong direction, and large positive values indicate rapid movement outwards. Thus, minute corrections that happen due to deviations from the reference trace of the spiral, for example resulting from subtle degradation of fine motor control, will result in increased variability in radial drawing velocity.

The notion that variability‐based features capture fine motor control is further supported by the partial correlations analysis. The statistically significant correlations with 9HPT after adjusting for oral SDMT and lack of correlation with oral SDMT after adjusting for 9HPT suggest that CV linear velocity of round shapes, CV angular velocity on the circle, and CV radial velocity on the spiral are primarily driven by a motor component.

The DaS Test differentiated PwMS from HC, with temporal and spatiotemporal features showing a stronger ability to differentiate PwMS from HC compared with spatial features. PwMS drew with increased drawing velocity variability and with reduced velocity and celerity than HC. Two features, CV linear velocity on the spiral and CV angular velocity on the circle, demonstrated the capacity to differentiate HC from PwMS with apparent normal upper extremity function (PwMS‐Normal). However, the lack of age‐ and sex‐controlled matching in this study limited our ability to adequately compare the HC and PwMS subgroups. We, therefore, used a global threshold to classify PwMS as normal or abnormal with respect to their upper extremity function. This threshold was derived from a normative population with a similar age and sex distribution as our PwMS cohort.^3^ An alternative approach to classify PwMS is to apply adaptive thresholds based on their age and sex. This methodology, however, is better suited for larger studies that enroll more diverse populations. Larger ongoing and future studies, including the CONSONANCE study (NCT03523858) and Floodlight™ MS – TONiC (ISRCTN11088592), will, therefore, explore the use of such adaptive thresholds.

We also noted differences in both analyses (correlations with standard clinical scales and brain MRI measures, and ability to differentiate between HC and PwMS) between individual shapes when considering the variability‐based velocity features. Complex, round shapes outperformed simple, linear shapes in both analyses. We suggest two possible explanations. First, it may become increasingly challenging for a demyelinated brain to quickly assemble the required motor plan as the complexity of the shapes increases. Second, complex, round shapes are characterized by a continuous and smooth change in trajectory, which may be challenging to complete for some PwMS. Drawing them may, therefore, rely more strongly on sensory feedback. Such feedback has been previously shown to play a role in the execution of visually guided tasks.^36^ Although complex shapes demonstrated the greatest sensitivity in this analysis, it is possible that simple shapes may become more relevant in patient populations with more significant impairment, especially if the patients struggle with drawing the complex shapes or have other comorbidities. Thus, including all shapes may enhance the test's versatility and comprehensiveness in assessing upper extremity function. Future analyses in a broader patient population, including in patients with progressive disease, will provide additional insights into the characteristics of each shape.

A few limitations and potential future study objectives are noted. First, PwMS enrolled in this study had mild, clinically stable disease in terms of both upper extremity dysfunction and overall disability, limiting the generalizability of the results. While the results are promising, including more PwMS‐Abnormal participants could help further validate the DaS Test. Second, our analyses were cross‐sectional given the relatively short study duration of 24 weeks and the stability of the standard clinical scales and MRI outcomes over the study period. This limited the ability to assess the DaS Test's sensitivity with regards to disease worsening or the utility to detect MS progression. Additionally, potentially relevant clinical correlates, for example, monofilament and grip strength testing were not investigated in this study. Future and ongoing studies, including the CONSONANCE and Floodlight MS – TONiC studies, shall clarify the use of the DaS Test in a larger, broader patient population (including people with more advanced disease), examine the test performance over time, derive composite measures from data, and outline the mapping of the features to a specific motor and sensory symptoms. Foundational work on establishing a composite, functional upper extremity score based on data derived from the DaS Test and other Floodlight tests has already been undertaken, showing good correlations with the 9HPT.^37^ These studies will also help to define normative bands for different levels of impairment and support the development of a digital upper extremity function score for easier interpretation of the performance on the DaS Test in clinical practice. Such a score could also be used as an endpoint in clinical research, for example, for drug development. Studies comprehensively assessing the correlation of multiple regional MRI outcomes with Floodlight test data and standard clinical measures are also underway.

In conclusion, the DaS Test provides an objective and self‐administered assessment of upper extremity function, which highlights its potential use in clinical practice and in clinical trials. We identified specific features derived from the DaS Test that correlated with measures of upper extremity dysfunction, the patient's perspective on the impact of the disease, and overall disease disability and severity, and that differentiated HC from PwMS. Further research is warranted in test‐specific symptomatology and in characterizing the DaS Test in wider patient populations and in other disease areas.

## Author Contributions

S. B., C. B., M. B., C. G., and M. L. designed and conceptualized the study. J. S. G. and X. M. contributed to participant enrollment and data collection. M. G. statistically analyzed the data. J. S. G., M. G., F. D., F. L., S. B., C. B., X. M., C. G., and M. L. interpreted the data. M. B. acquired funding for the study. All authors critically reviewed and approved the final version of the manuscript.

## Conflicts of Interest

The authors disclosed receipt of the following financial support for the research, authorship, and/or publication of this article: F. Hoffmann‐La Roche Ltd, Basel, Switzerland, provided financial support for the study and publication of this manuscript. J. S. Graves has received research support from Biogen, EMD Serono, Novartis, and Sanofi; has received speaking honoraria from Bristol Myers Squibb, Bayer, and Alexion; and served on advisory boards for Genentech and Bayer. M. Ganzetti is a contractor for F. Hoffmann‐La Roche Ltd. F. Dondelinger was an employee of and is a shareholder in F. Hoffmann‐La Roche Ltd; he is currently employed by Novartis Institutes for Biomedical Research. F. Lipsmeier is an employee of F. Hoffmann‐La Roche Ltd. S. Belachew was an employee of F. Hoffmann‐La Roche Ltd during the completion of the work related to this manuscript and is now an employee of Biogen (Cambridge, MA), which is not in any way associated with this study. C. Bernasconi is a contractor for F. Hoffmann‐La Roche Ltd. X. Montalban has received speaking honoraria and travel expenses for participation in scientific meetings; has been a steering committee member of clinical trials or participated in advisory boards of clinical trials in the past years with Actelion, Alexion, Bayer, Biogen, Celgene, EMD Serono, Genzyme, Immunic, MedDay, Merck, Mylan, Nervgen, Novartis, Roche, Sanofi‐Genzyme, Teva Pharmaceuticals, TG Therapeutics, Excemed, MSIF, and NMSS. J. van Beek was an employee of F. Hoffmann‐La Roche Ltd during the completion of the work related to this manuscript and is now an employee of Biogen (Cambridge, MA), which is not in any way associated with this study. M. Baker and C. Gossens are employees of and shareholders in F. Hoffmann‐La Roche Ltd. M. Lindemann is a consultant to F. Hoffmann‐La Roche Ltd via Inovigate.

## Supporting information


**Figure S1.** The Draw a Shape Test features.
**Figure S2.** Clinical and MRI measures from baseline to week 24 (end of study).
**Figure S3.** Increased variability in spiral radial velocity with higher levels of MS‐related impairment.
**Table S1.** Spearman's rank correlations with standard clinical scales and MRI measures in PwMS by handedness.
**Table S2.** Descriptive statistics by subject group and handedness.

## Data Availability

For up‐to‐date details on Roche's Global Policy on Sharing of Clinical Study Information and how to request access to related clinical study documents, see https://go.roche.com/data_sharing. Request for the data underlying this publication requires a detailed, hypothesis‐driven statistical analysis plan that is collaboratively developed by the requestor and company subject matter experts. Such requests should be directed to dbm.datarequest@roche.com for consideration. Anonymized records for individual patients across more than one data source external to Roche cannot, and should not, be linked due to a potential increase in the risk of patient re‐identification.
