# Supplementary material for: Preliminary validity of the Draw a Shape Test for upper extremity assessment in multiple sclerosis
Source: Ann Clin Transl Neurol. 2022 Dec 23;10(2):166–80. doi: 10.1002/acn3.51705 (PMC9930424; doi:10.1002/acn3.51705)
Supplement: Supplementary file 1 — Figure S1. The Draw a Shape Test features. Figure S2. Clinical and MRI measures from baseline to week 24 (end of study). Figure S3. Increased variability in spiral radial velocity with higher levels of MS‐related impairment. Table S1. Spearman's rank correlations with standard clinical scales and MRI measures in PwMS by handedness. Table S2. Descriptive statistics by subject group and handedness. [file ACN3-10-166-s001.pdf]

Supplementary Appendix

Preliminary validity of the Draw a Shape Test for upper extremity assessment in multiple sclerosis

Jennifer S. Graves, MAS, MD, PhD[1], Marco Ganzetti, PhD[2], Frank Dondelinger, PhD[2]*, Florian Lipsmeier, PhD[2], Shibeshih Belachew, MD, PhD[2], Corrado Bernasconi, MD, PhD[2], Xavier Montalban, MD, PhD[3], Johan van Beek, PhD[2]**, Michael Baker, MSc[2], Christian Gossens, MBA, PhD[2] & Michael Lindemann, PhD[2]

[1]Department of Neurosciences, University of California San Diego, San Diego, CA, USA

[2]F. Hoffmann-La Roche Ltd, Basel, Switzerland

[3]Department of Neurology-Neuroimmunology, Centre d'Esclerosi Múltiple de Catalunya (Cemcat), Hospital Universitari Vall d'Hebron, Barcelona, Spain

*At the time of writing author was an employee of F. Hoffmann-La Roche Ltd; current affiliation is Novartis Institutes for Biomedical Research, Basel, Switzerland

**At the time of writing author was an employee of F. Hoffmann-La Roche Ltd; current affiliation is Biogen, Cambridge, MA, US

A

Feature	Computational definition	Variables
**Spatial features**		
**Trace accuracy**	$DT \cap RT$	DT: touch points of the drawn trace<br><br>RT: reference shape trace
**Temporal features**		
**Mean linear velocity, mm/s**	$mean(\dot{y}(t_n))$	$\dot{y}(t_n) = \frac{dy(t)}{dt}\Big\vert_{t=t_n}$: derivative of the displacement (mm/s)<br>$y(t)$ *:* the displacement time series as a function of time
**CV linear velocity**	$\frac{SD(\dot{y}(t_n))}{mean(\dot{y}(t_n))}$	$\dot{y}(t_n) = \frac{dy(t)}{dt}\Big\vert_{t=t_n}$: derivative of the displacement (mm/s)<br>$y(t)$ *:* the displacement time series as a function of time
**Mean angular velocity, rad/s**	$mean\left(\dot{\Theta}(t_n)\right)$	$\dot{\Theta}(t_n) = \frac{d\Theta(t)}{dt}\Big\vert_{t=t_n}$: derivative of the angular displacement (rad/s)<br>$\Theta(t)$ *:* the angular displacement time series as a function of time
**CV angular velocity**	$\frac{SD\left(\dot{\Theta}(t_n)\right)}{mean\left(\dot{\Theta}(t_n)\right)}$	$\dot{\Theta}(t_n) = \frac{d\Theta(t)}{dt}\Big\vert_{t=t_n}$: derivative of the angular displacement (rad/s)<br>$\Theta(t)$ *:* the angular displacement time series as a function of time
**Mean radial velocity, mm/s**	$mean(\dot{r}(t_n))$	$\dot{r}(t_n) = \frac{dr(t)}{dt}\Big\vert_{t=t_n}$: derivative of the radial displacement (mm/s)<br>$r(t)$ *:* the radial displacement time series as a function of time
**CV radial velocity**	$\frac{SD(\dot{r}(t_n))}{mean(\dot{r}(t_n))}$	$\dot{r}(t_n) = \frac{dr(t)}{dt}\Big\vert_{t=t_n}$: derivative of the radial displacement (mm/s)<br>$r(t)$ *:* the radial displacement time series as a function of time
**Dwell time ratio**	$\frac{\sum_{i=1}^{n} t_i}{D_T}$	$\sum_{i=1}^{n} t_i$: total time spent at the corners (*n* = 3) of the SQUARE shape, computed as the full width at half minimum of the troughs in the velocity time series (s)<br><br>D_T: total drawing time (s)
**Spatiotemporal features**		
**Trace celerity, 1/s**	$\frac{Trace\ accuracy}{D_T}$	D_T: total drawing time (s)

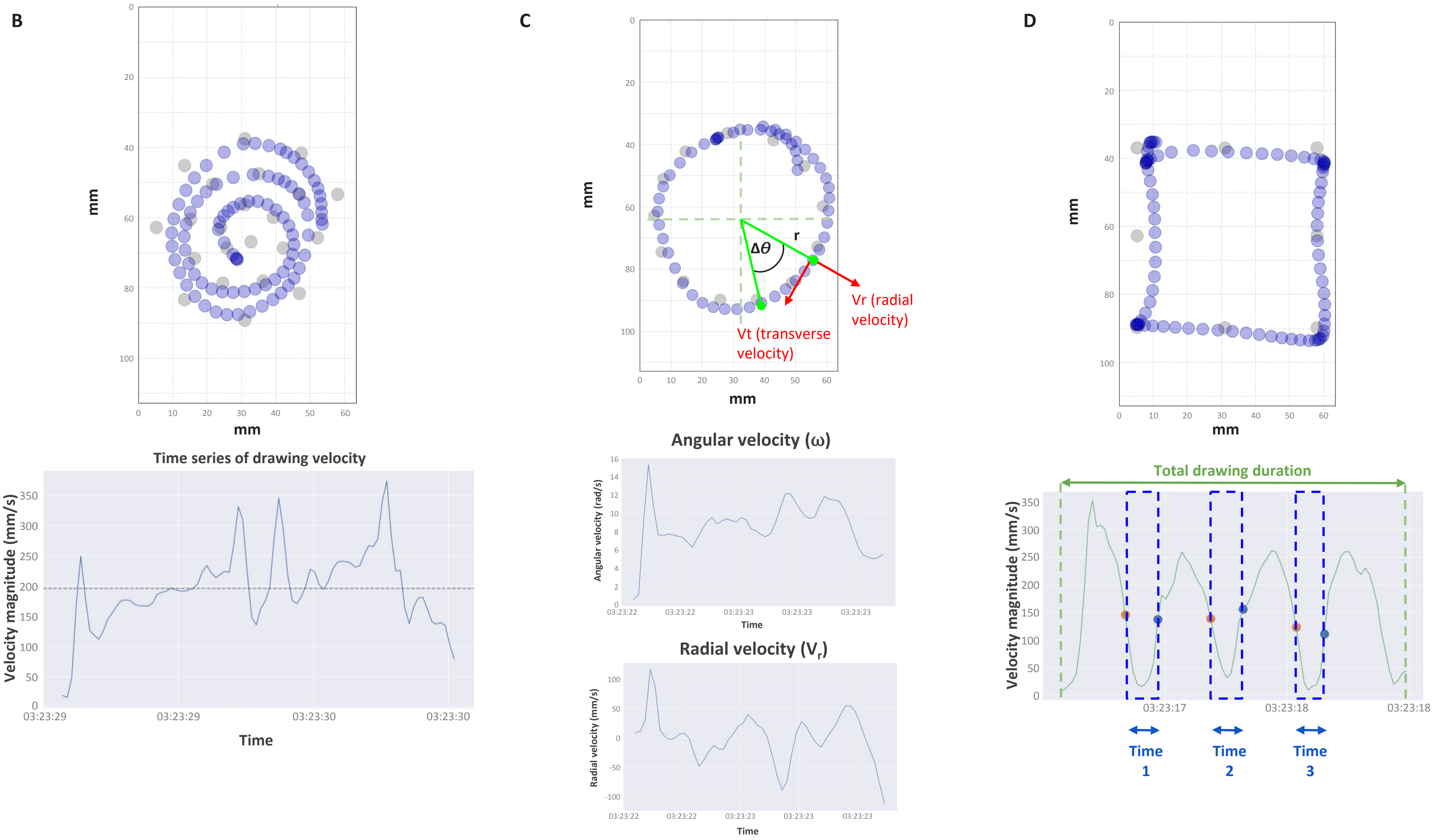

Supplementary figure 1. The Draw a Shape Test features.
(A) Mathematical definitions for each of the feature groups. (B) Illustration of linear velocity with drawn trace (top) and time series (bottom). Linear velocity was computed as either the mean or the coefficient of variation (CV) over time for all shapes individually. (C) Illustration of angular and radial velocities with drawn trace (top) and time series (bottom). Angular and radial velocity features were computed as either the mean or the CV over time for the circle and spiral separately. (D) Illustration of dwell time ratio with drawn trace (top) and time series (bottom). Dwell time ratio is defined as the ratio of the time spent in the three corners of the square, computed as the full width at half minimum of the troughs in the velocity time series, divided by the total drawing duration measured in seconds. This feature was computed for the square only.

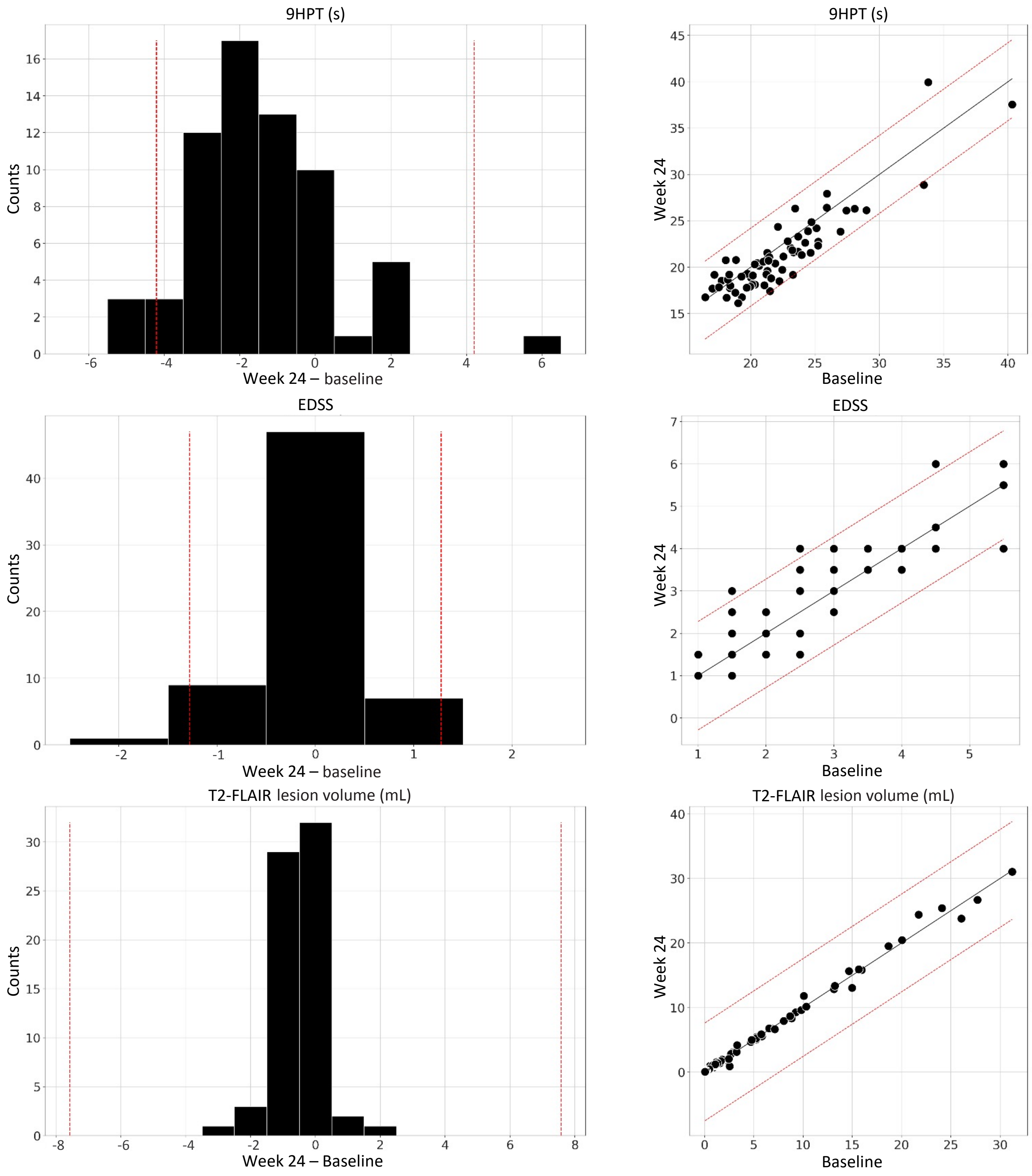

Supplementary figure 2. Clinical and MRI measures from baseline to week 24 (end of study).
Histograms (left) depicting the changes of 9HPT (top row), EDSS (middle row), and T2-FLAIR lesion volume (bottom row) from baseline to week 24. Scatter plots (right) showing relationship of each measure at baseline and week 24. The red-dashed lines indicate the standard deviation of the 9HPT, EDSS, and T2-FLAIR lesion volume measurements at baseline.
9HPT, Nine-Hole Peg Test; EDSS, Expanded Disability Status Scale; FLAIR, fluid-attenuated inversion recovery.

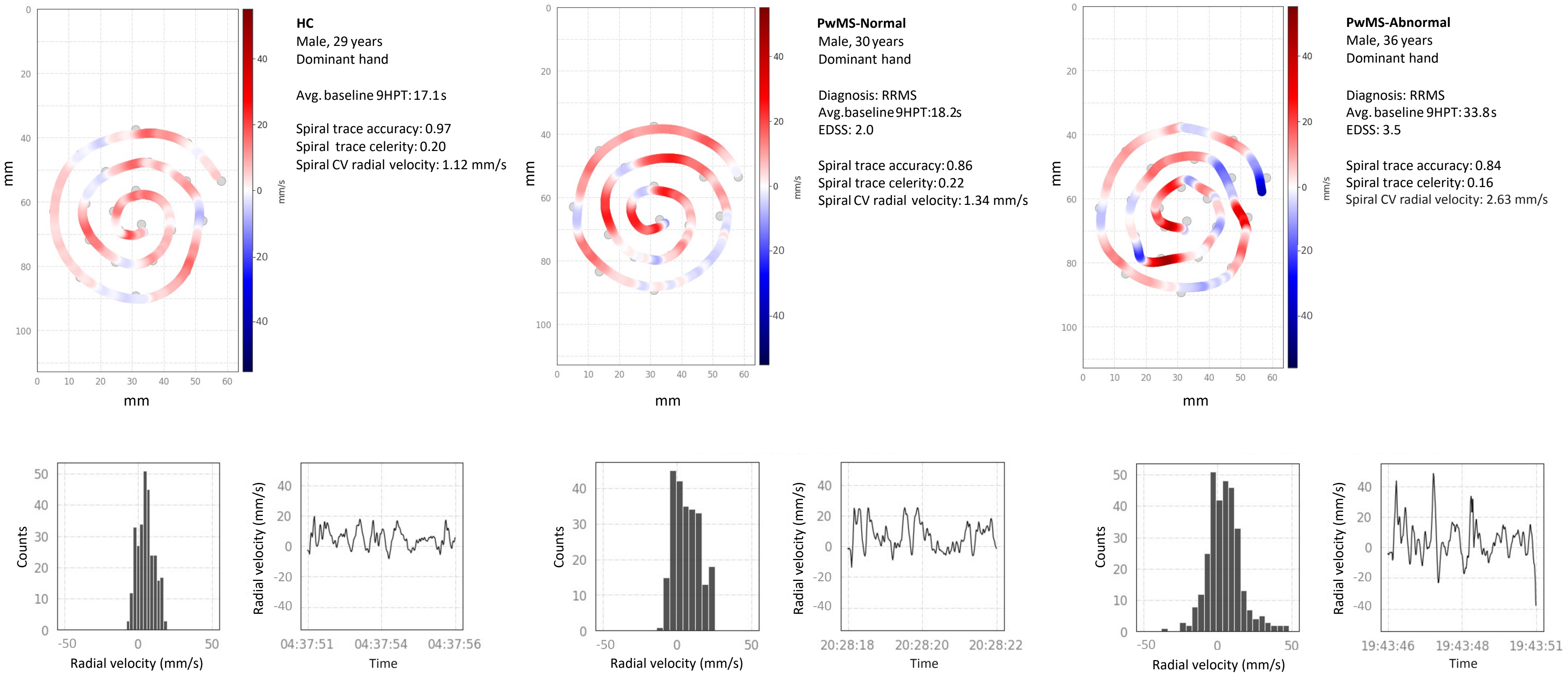

Supplementary figure 3. Increased variability in spiral radial velocity with higher levels of MS-related impairment.
The top row shows the drawn traces of the spiral shape for a HC (left), a PwMS-Normal (middle), and a PwMS-Abnormal (right), with the color bar indicating the radial velocity. The bottom row shows the corresponding histograms and time series of the spiral radial velocity.
9HPT, Nine-Hole Peg Test; CV, coefficient of variation; EDSS, Expanded Disability Status Scale; HC, healthy control; MS, multiple sclerosis; PwMS, people with multiple sclerosis; RRMS, relapsing-remitting multiple sclerosis.

Supplementary table 1. Spearman's rank correlations with standard clinical scales and MRI measures in PwMS by handedness.

Feature	Handedness	Spearman's rank correlation coefficient[1]						
		9HPT, s	MSIS-29 hand items	EDSS	EDSS cerebellar functional system	EDSS pyramidal functional system	Total brain volume, mL	T2-lesion volume, mL
9HPT time, s	D	**1.00**	**0.50**	**0.57**	**0.65**	**0.44**	**-0.45**	0.18
	ND	**1.00**	**0.37**	**0.43**	**0.54**	**0.36**	**-0.41**	**0.32**
**Spatial features**								
Trace accuracy								
Line bottom to top	D	**-0.33**	**-0.36**	**-0.36**	-0.16	**-0.36**	**0.29**	-0.19
	ND	**-0.38**	**-0.30**	**-0.36**	**-0.26**	**-0.32**	**0.39**	**-0.34**
Line top to bottom	D	**-0.30**	**-0.32**	**-0.38**	-0.23	**-0.38**	**0.29**	-0.14
	ND	**-0.26**	**-0.34**	**-0.41**	-0.23	**-0.37**	**0.41**	-0.24
Square	D	**-0.32**	**-0.37**	**-0.36**	-0.23	**-0.34**	**0.32**	-0.20
	ND	**-0.44**	**-0.42**	**-0.43**	**-0.38**	**-0.38**	**0.46**	**-0.25**
Circle	D	**-0.36**	**-0.38**	**-0.37**	-0.20	**-0.35**	0.24	-0.15
	ND	**-0.41**	**-0.36**	**-0.40**	**-0.26**	**-0.37**	**0.37**	**-0.27**
Figure-of-8	D	**-0.44**	**-0.43**	**-0.38**	**-0.25**	**-0.34**	0.23	-0.16
	ND	**-0.42**	**-0.35**	**-0.33**	-0.22	**-0.30**	**0.37**	**-0.26**
Spiral	D	**-0.38**	**-0.45**	**-0.39**	-0.22	**-0.32**	**0.27**	-0.24
	ND	**-0.49**	**-0.38**	**-0.39**	**-0.27**	**-0.31**	**0.42**	**-0.29**
Mean overall trace accuracy	D	**-0.38**	**-0.43**	**-0.41**	-0.22	**-0.39**	**0.27**	-0.21
	ND	**-0.42**	**-0.40**	**-0.44**	**-0.28**	**-0.40**	**0.43**	**-0.30**
**Temporal features**								
Mean linear velocity, mm/s								
Line bottom to top	D	-0.11	0.21	0.12	-0.18	0.16	0.10	-0.17
	ND	-0.19	0.18	0.19	-0.03	0.17	0.01	-0.04
Line top to bottom	D	-0.20	0.11	0.00	**-0.25**	0.03	0.17	-0.21
	ND	-0.24	0.17	0.12	-0.07	0.15	0.05	-0.01
Square	D	-0.15	0.16	0.08	-0.19	0.09	0.10	-0.12
	ND	**-0.24**	0.12	0.13	-0.04	0.12	0.00	-0.08
Circle	D	-0.12	0.17	0.12	-0.18	0.15	0.11	-0.11
	ND	-0.17	0.20	0.15	-0.08	0.14	0.02	-0.01
Figure-of-8	D	-0.14	0.15	0.06	-0.22	0.09	0.17	-0.13
	ND	-0.17	0.18	0.13	-0.10	0.14	0.04	0.00
Spiral	D	-0.13	0.17	0.07	-0.23	0.08	0.17	-0.13
	ND	-0.20	0.20	0.17	-0.08	0.15	0.01	0.01

CV linear velocity								
Line bottom to top	D	0.09	0.13	0.10	-0.06	0.14	-0.09	-0.01
	ND	-0.09	-0.16	0.02	-0.04	0.05	-0.24	**0.26**
Line top to bottom	D	0.18	0.12	0.15	0.10	0.18	-0.11	0.00
	ND	0.13	-0.02	0.10	0.06	0.06	**-0.28**	0.21
Square	D	0.22	0.15	0.15	0.13	0.13	-0.16	0.21
	ND	0.21	0.06	0.15	0.14	0.16	**-0.33**	**0.28**
Circle	D	**0.38**	0.19	**0.31**	**0.39**	**0.35**	**-0.40**	**0.25**
	ND	**0.47**	0.06	0.13	**0.28**	0.20	**-0.44**	**0.32**
Figure-of-8	D	**0.52**	**0.35**	**0.45**	**0.47**	**0.49**	**-0.35**	**0.35**
	ND	**0.55**	0.15	**0.28**	**0.32**	**0.31**	**-0.36**	**0.31**
Spiral	D	**0.48**	**0.42**	**0.39**	**0.41**	**0.27**	**-0.29**	**0.35**
	ND	**0.46**	0.18	0.18	**0.31**	0.13	**-0.37**	**0.39**
Mean angular velocity, rad/s								
Circle	D	-0.14	0.17	0.12	-0.19	0.14	0.12	-0.11
	ND	-0.17	0.20	0.16	-0.06	0.15	0.02	-0.01
Spiral	D	-0.15	0.14	0.05	**-0.25**	0.05	0.18	-0.13
	ND	-0.23	0.17	0.14	-0.10	0.13	0.06	-0.04
CV angular velocity								
Circle	D	**0.38**	0.15	**0.27**	**0.41**	**0.29**	**-0.40**	**0.24**
	ND	**0.48**	0.05	0.11	**0.27**	0.17	**-0.44**	**0.34**
Spiral	D	**0.27**	0.12	0.15	0.19	0.10	-0.15	0.21
	ND	**0.47**	0.13	**0.31**	**0.34**	0.23	-0.24	0.22
Mean radial velocity, mm/s								
Circle	D	0.14	0.11	0.15	-0.01	0.16	-0.17	-0.07
	ND	-0.04	-0.11	-0.13	-0.03	-0.15	-0.05	0.09
Spiral	D	-0.16	0.15	0.06	-0.22	0.06	0.19	-0.13
	ND	**-0.26**	0.16	0.12	-0.11	0.10	0.07	-0.03
CV radial velocity								
Circle	D	0.20	0.02	0.11	0.19	0.07	-0.13	-0.05
	ND	0.12	-0.14	-0.08	0.02	-0.14	-0.11	0.08
Spiral	D	**0.66**	**0.54**	**0.53**	**0.49**	**0.45**	**-0.39**	**0.28**
	ND	**0.61**	**0.35**	**0.45**	**0.41**	**0.39**	**-0.39**	**0.33**
Dwell time ratio								
Square	D	0.17	**0.30**	0.17	0.03	0.15	-0.15	0.24
	ND	0.17	**0.25**	0.22	0.13	0.22	-0.23	**0.25**

**Spatiotemporal features**								
Trace celerity, 1/s								
Line bottom to top	D	**-0.26**	0.10	-0.05	**-0.28**	0.00	**0.25**	**-0.31**
	ND	**-0.39**	0.12	0.02	-0.19	0.05	0.15	-0.15
Line top to bottom	D	**-0.37**	-0.02	-0.21	**-0.39**	-0.15	**0.34**	**-0.32**
	ND	**-0.42**	-0.02	-0.06	-0.16	-0.04	0.21	-0.12
Square	D	**-0.30**	0.05	-0.09	**-0.34**	-0.04	**0.26**	**-0.25**
	ND	**-0.47**	-0.03	-0.05	-0.17	-0.03	0.21	-0.17
Circle	D	**-0.31**	0.05	-0.03	**-0.28**	0.01	**0.26**	-0.20
	ND	**-0.43**	0.03	-0.05	-0.21	-0.03	0.19	-0.18
Figure-of-8	D	**-0.37**	-0.01	-0.13	**-0.36**	-0.07	**0.31**	**-0.26**
	ND	**-0.48**	0.00	-0.02	-0.16	0.01	**0.25**	-0.14
Spiral	D	**-0.31**	-0.01	-0.13	**-0.38**	-0.09	**0.32**	-0.23
	ND	**-0.49**	0.03	-0.05	**-0.24**	-0.03	**0.26**	-0.23
Mean overall trace celerity	D	**-0.33**	0.04	-0.12	**-0.35**	-0.07	**0.30**	**-0.29**
	ND	**-0.45**	0.06	-0.03	-0.20	0.01	0.22	-0.15

Higher values indicated better performance on features assessing trace accuracy, mean velocity, and trace celerity. Lower values indicated better performance on features assessing CV velocity and dwell time ratio.

9HPT, Nine-Hole Peg Test; CV, coefficient of variation; D, dominant hand; EDSS, Expanded Disability Status Scale; ND, nondominant hand; PwMS, people with multiple sclerosis.

[1]Spearman's rank correlation coefficients highlighted in bold face are statistically significant at $p < 0.05$.

Supplementary table 2. Descriptive statistics by subject group and handedness.

		Mean ± SD				p[1]			
**Feature**	**Handedness**	**HC (*n* = 18 for D; *n* = 18 for ND)**	**PwMS (*n* = 69 for D; *n* = 59 for ND)**	**PwMS-Normal (*n* = 47 for D; *n* = 49 for ND)**	**PwMS-Abnormal (*n* = 22 for D; *n* = 20 for ND)**	**HC vs. PwMS**	**HC vs. PwMS-Low**	**HC vs. PwMS-Abnormal**	**PwMS-Low vs. PwMS-Abnormal**
**Spatial features**									
Trace accuracy									
Line bottom	D	0.90 ± 0.08	0.87 ± 0.11	0.88 ± 0.12	0.83 ± 0.11	0.368	0.837	0.050	0.080
to top	ND	0.94 ± 0.09	0.92 ± 0.11	0.95 ± 0.10	0.87 ± 0.11	0.543	0.888	**0.047**	**0.006**
Line top to	D	0.85 ± 0.06	0.81 ± 0.12	0.82 ± 0.11	0.77 ± 0.12	0.351	0.781	0.057	0.067
bottom	ND	0.91 ± 0.07	0.89 ± 0.10	0.91 ± 0.10	0.83 ± 0.09	0.516	0.734	**0.012**	**0.003**
Square	D	0.91 ± 0.05	0.89 ± 0.09	0.90 ± 0.08	0.87 ± 0.10	0.637	1.000	0.221	0.315
	ND	0.92 ± 0.07	0.89 ± 0.10	0.92 ± 0.09	0.84 ± 0.09	0.346	0.799	**0.002**	**<0.001**
Circle	D	0.94 ± 0.05	0.91 ± 0.09	0.92 ± 0.09	0.88 ± 0.11	0.463	0.815	0.142	0.149
	ND	0.90 ± 0.08	0.87 ± 0.11	0.90 ± 0.10	0.80 ± 0.10	0.432	0.682	**0.002**	**<0.001**
Figure-of-8	D	0.94 ± 0.05	0.90 ± 0.10	0.92 ± 0.09	0.87 ± 0.10	0.216	0.681	**0.014**	**0.034**
	ND	0.93 ± 0.09	0.90 ± 0.12	0.94 ± 0.10	0.82 ± 0.11	0.476	0.641	**0.003**	**<0.001**
Spiral	D	0.89 ± 0.05	0.85 ± 0.10	0.87 ± 0.08	0.82 ± 0.12	0.232	0.598	**0.034**	0.062
	ND	0.89 ± 0.07	0.86 ± 0.09	0.89 ± 0.08	0.79 ± 0.10	0.356	0.832	**0.003**	**<0.001**
Mean overall	D	0.88 ± 0.04	0.85 ± 0.09	0.87 ± 0.09	0.83 ± 0.10	0.314	0.681	0.065	0.099
trace accuracy	ND	0.91 ± 0.07	0.88 ± 0.09	0.91 ± 0.08	0.82 ± 0.09	0.373	0.788	**0.002**	**<0.001**
**Temporal features**									
Mean linear velocity, mm/s									
Line bottom	D	62.7 ± 19.3	55.0 ± 33.9	55.4 ± 21.4	54.0 ± 52.2	0.075	0.247	**0.013**	0.050
to top	ND	39.9 ± 15.1	35.0 ± 31.6	38.3 ± 35.9	27.0 ± 14.9	**0.029**	0.090	**0.010**	0.132
Line top to	D	61.7 ± 19.7	55.2 ± 32.0	56.9 ± 20.4	51.4 ± 48.9	0.078	0.333	**0.006**	**0.009**
bottom	ND	34.7 ± 12.7	33.6 ± 28.3	36.6 ± 31.1	26.2 ± 18.5	0.368	0.788	0.050	0.062
Square	D	58.1 ± 9.9	50.0 ± 19.4	52.2 ± 14.4	45.3 ± 26.9	**0.024**	0.139	**0.002**	**0.012**
	ND	39.9 ± 7.8	34.6 ± 19.1	37.3 ± 20.3	27.9 ± 13.9	**0.035**	0.174	**0.002**	**0.038**
Circle	D	51.7 ± 11.7	46.1 ± 24.3	47.5 ± 15.5	43.2 ± 37.1	0.065	0.305	**0.004**	**0.017**
	ND	33.0 ± 8.2	30.5 ± 24.3	33.2 ± 26.7	24.0 ± 15.6	0.084	0.264	**0.012**	0.083
Figure-of-8	D	44.9 ± 9.1	38.1 ± 21.2	39.2 ± 13.7	35.7 ± 32.1	**0.008**	**0.045**	**0.001**	**0.028**
	ND	27.5 ± 6.7	24.0 ± 21.0	26.6 ± 23.3	17.6 ± 12.1	**0.031**	0.098	**0.009**	0.057
Spiral	D	50.8 ± 9.7	45.0 ± 23.3	45.8 ± 15.0	43.1 ± 35.5	**0.019**	0.095	**0.003**	**0.028**
	ND	33.1 ± 7.3	30.2 ± 22.0	33.0 ± 23.9	23.4 ± 14.5	**0.041**	0.166	**0.005**	**0.044**

CV linear velocity									
Line bottom to top	D	0.51 ± 0.07	0.53 ± 0.09	0.52 ± 0.09	0.53 ± 0.09	0.683	0.681	0.765	0.898
	ND	0.43 ± 0.06	0.47 ± 0.08	0.47 ± 0.08	0.46 ± 0.10	0.075	**0.042**	0.447	0.500
Line top to bottom	D	0.51 ± 0.08	0.53 ± 0.10	0.51 ± 0.10	0.56 ± 0.11	0.714	0.860	0.201	0.139
	ND	0.45 ± 0.06	0.49 ± 0.09	0.48 ± 0.09	0.50 ± 0.08	0.197	0.365	0.085	0.302
Square	D	0.55 ± 0.06	0.57 ± 0.10	0.55 ± 0.08	0.60 ± 0.13	0.600	0.895	0.265	0.189
	ND	0.49 ± 0.06	0.52 ± 0.08	0.51 ± 0.07	0.55 ± 0.09	0.271	0.641	**0.035**	**0.033**
Circle	D	0.31 ± 0.02	0.33 ± 0.04	0.32 ± 0.03	0.36 ± 0.05	**0.018**	0.177	**<0.001**	**0.002**
	ND	0.36 ± 0.03	0.40 ± 0.05	0.38 ± 0.04	0.44 ± 0.06	**0.004**	**0.045**	**<0.001**	**<0.001**
Figure-of-8	D	0.34 ± 0.02	0.36 ± 0.04	0.35 ± 0.03	0.38 ± 0.06	0.080	0.519	**<0.001**	**0.001**
	ND	0.37 ± 0.03	0.40 ± 0.05	0.39 ± 0.04	0.45 ± 0.06	**0.041**	0.420	**<0.001**	**<0.001**
Spiral	D	0.31 ± 0.03	0.35 ± 0.05	0.33 ± 0.03	0.38 ± 0.07	**<0.001**	**0.015**	**<0.001**	**<0.001**
	ND	0.38 ± 0.05	0.43 ± 0.06	0.41 ± 0.04	0.47 ± 0.07	**0.004**	**0.048**	**<0.001**	**<0.001**
Mean angular velocity, rad/s									
Circle	D	1.93 ± 0.45	1.73 ± 0.94	1.78 ± 0.59	1.63 ± 1.45	0.070	0.326	**0.004**	**0.016**
	ND	1.24 ± 0.30	1.15 ± 0.92	1.25 ± 1.02	0.90 ± 0.59	0.080	0.258	**0.011**	0.086
Spiral	D	3.06 ± 0.61	2.71 ± 1.42	2.77 ± 0.92	2.59 ± 2.16	**0.020**	0.107	**0.002**	**0.022**
	ND	1.97 ± 0.39	1.83 ± 1.35	2.02 ± 1.48	1.37 ± 0.83	**0.039**	0.198	**0.002**	**0.019**
CV angular velocity									
Circle	D	0.30 ± 0.02	0.33 ± 0.05	0.31 ± 0.03	0.35 ± 0.06	**0.015**	0.124	**<0.001**	**0.002**
	ND	0.36 ± 0.03	0.40 ± 0.06	0.38 ± 0.04	0.45 ± 0.07	**0.003**	**0.039**	**<0.001**	**<0.001**
Spiral	D	0.54 ± 0.04	0.53 ± 0.06	0.53 ± 0.05	0.54 ± 0.07	0.958	0.837	0.605	0.309
	ND	0.55 ± 0.05	0.55 ± 0.07	0.54 ± 0.06	0.59 ± 0.08	0.967	0.351	0.070	**0.003**
Mean radial velocity, mm/s									
Circle	D	0.02 ± 0.27	0.02 ± 0.31	-0.01 ± 0.24	0.09 ± 0.43	0.489	0.509	0.568	0.857
	ND	0.10 ± 0.32	0.18 ± 0.38	0.21 ± 0.40	0.12 ± 0.34	0.777	0.724	0.953	0.751
Spiral	D	4.34 ± 0.84	3.79 ± 1.86	3.89 ± 1.21	3.57 ± 2.82	**0.017**	0.086	**0.003**	**0.020**
	ND	2.93 ± 0.55	2.65 ± 1.79	2.93 ± 1.93	1.98 ± 1.15	**0.021**	0.134	**<0.001**	**0.009**
CV radial velocity									
Circle	D	0.79 ± 10.91	1.39 ± 8.85	0.82 ± 8.74	2.59 ± 9.16	0.550	0.379	0.935	0.328
	ND	13.86 ± 11.02	13.65 ± 12.29	12.99 ± 12.67	15.28 ± 11.46	0.536	0.724	0.320	0.355
Spiral	D	1.71 ± 0.16	1.87 ± 0.39	1.74 ± 0.24	2.16 ± 0.05	0.241	0.815	**<0.001**	**<0.001**
	ND	2.23 ± 0.29	2.43 ± 0.58	2.22 ± 0.37	2.94 ± 0.67	0.402	0.488	**<0.001**	**<0.001**
Dwell time ratio									
Square	D	0.39 ± 0.04	0.37 ± 0.07	0.37 ± 0.06	0.38 ± 0.07	0.483	0.403	0.786	0.662
	ND	0.39 ± 0.05	0.39 ± 0.06	0.38 ± 0.05	0.42 ± 0.07	0.942	0.534	0.279	0.059

**Spatiotemporal features**									
Trace celerity, s^{-1}									
Line bottom to top	D	0.82 ± 0.23	0.67 ± 0.33	0.70 ± 0.23	0.61 ± 0.48	**0.013**	0.074	**0.002**	**0.009**
	ND	0.59 ± 0.16	0.52 ± 0.30	0.58 ± 0.33	0.38 ± 0.16	**0.049**	0.302	**<0.001**	**0.002**
Line top to bottom	D	0.70 ± 0.20	0.58 ± 0.31	0.63 ± 0.21	0.48 ± 0.44	**0.037**	0.278	**<0.001**	**<0.001**
	ND	0.46 ± 0.13	0.43 ± 0.27	0.49 ± 0.29	0.28 ± 0.14	0.167	0.944	**<0.001**	**<0.001**
Square	D	0.25 ± 0.04	0.20 ± 0.06	0.22 ± 0.05	0.18 ± 0.06	**0.002**	**0.014**	**<0.001**	**0.003**
	ND	0.17 ± 0.03	0.15 ± 0.06	0.16 ± 0.05	0.11 ± 0.04	**0.007**	0.092	**<0.001**	**<0.001**
Circle	D	0.28 ± 0.05	0.24 ± 0.07	0.25 ± 0.05	0.21 ± 0.09	**0.012**	0.110	**<0.001**	**0.001**
	ND	0.19 ± 0.03	0.16 ± 0.06	0.18 ± 0.06	0.12 ± 0.04	**0.010**	0.179	**<0.001**	**<0.001**
Figure-of-8	D	0.30 ± 0.05	0.24 ± 0.08	0.26 ± 0.06	0.21 ± 0.10	**0.002**	**0.022**	**<0.001**	**<0.001**
	ND	0.20 ± 0.03	0.17 ± 0.07	0.20 ± 0.07	0.12 ± 0.04	**0.004**	0.110	**<0.001**	**<0.001**
Spiral	D	0.16 ± 0.03	0.13 ± 0.04	0.14 ± 0.03	0.12 ± 0.06	**0.003**	**0.030**	**<0.001**	**0.002**
	ND	0.11 ± 0.01	0.09 ± 0.04	0.10 ± 0.04	0.07 ± 0.03	**0.006**	0.110	**<0.001**	**<0.001**
Mean overall trace celerity	D	0.42 ± 0.08	0.35 ± 0.14	0.37 ± 0.10	0.30 ± 0.19	**0.009**	0.071	**<0.001**	**0.002**
	ND	0.29 ± 0.05	0.26 ± 0.12	0.29 ± 0.13	0.18 ± 0.07	**0.026**	0.302	**<0.001**	**<0.001**

Higher values indicated better performance on features assessing trace accuracy, mean velocity, and trace celerity. Lower values indicated better performance on features assessing CV velocity and dwell time ratio. *p* values in bold are < 0.05.

CV, coefficient of variation; D, dominant hand; HC, healthy control; ND, nondominant hand; PwMS, people with multiple sclerosis.

[1]Mann–Whitney U test.
